# *MMP8* polymorphism is associated with susceptibility to osteonecrosis of the femoral head in a Chinese Han population

**DOI:** 10.18632/oncotarget.15371

**Published:** 2017-02-16

**Authors:** Feimeng An, Jieli Du, Yuju Cao, Jianping Shi, Yongchang Guo, Tianbo Jin, Jian Li, Junyu Chen, Ping Li, Mei Dong, Guoqiang Wang, Jianzhong Wang

**Affiliations:** ^1^ Inner Mongolia Medical University, Hohhot, Inner Mongolia, China; ^2^ Department of Orthopedics and Traumatology, The Second Affiliated Hospital of Inner Mongolia University, Hohhot, Inner Mongolia, China; ^3^ Zhengzhou TCM Traumatology Hospital, Zhengzhou, Henan, China; ^4^ Department of TCM Diagnosis, Inner Mongolia Medical University, Hohhot, China; ^5^ MOE Key Laboratory of Resource Biology and Modern Biotechnology, Northwest University, Xi’an, China

**Keywords:** MMP8, single nucleotide polymorphisms, osteonecrosis of the femoral head, association study

## Abstract

Osteonecrosis of the femoral head (ONFH) is an orthopedic refractory disease that adversely affects quality of life. Matrix metalloproteinase-8 (MMP-8) produced by the bone marrow has been implicated in the degradation of collagen during bone development. We assessed whether *MMP8* polymorphisms are associated with ONFH. In a case-control study, using χ^2^ tests and genetic model analyses, we genotyped 5 *MMP8* single-nucleotide polymorphisms (SNPs) in 585 ONFH patients and 507 healthy control subjects in a Chinese Han population. The *MMP8* rs11225394 SNP was associated with an increased risk of ONFH in an allele model (OR=1.34; 95% CI, 1.003-1.786, P=0.047). In addition, rs11225394 was associated with an increased risk of ONFH in a dominant model (OR =1.39, 95% CI, 1.02–1.89, P=0.036), over-dominant model (OR=1.39, 95% CI, 1.02–1.89, P=0.038), and log-additive model (OR =1.36, 95% CI, 1.01–1.84, P=0.039). After adjusting for age and gender, rs11225394 was associated with ONFH in a dominant (OR =1.44, 95% CI, 1.05–1.96, P=0.023), over-dominant (OR =1.44, 95% CI, 1.05–1.98, P=0.022), and log-additive model (OR =1.40, 95% CI, 1.04–1.90, P=0.027). These results provide the first evidence that *MMP8* SNP at the rs11225394 locus is associated with the increased risk of ONFH in Chinese Han population.

## INTRODUCTION

Osteonecrosis of the femoral head (ONFH) is an intractable bone disease resulting in considerable loss of function of common hip joint. Without treatment, osteonecrosis usually progresses to femoral head collapse and subsequent degenerative arthritis of the hip joint [1]. Despite preserving and surgical treatments, morbidity and disability from osteonecrosis in the Chinese Han population continue to rise. Therefore, early detection is crucial to improve outcomes and patients quality of life. Since early detection of ONFH remains difficult due to the lack of early clinical symptoms, there is an urgent need to elucidate the etiology and pathogenic mechanisms underlying ONFH.

Bone marrow mesenchymal stem cells (BMSCs) have the potential to differentiate into osteoblasts of bone-building, and osteoclasts of bone-destroying. In general, the amount of bone removed by the osteoclasts, and the amount of bone formed by the osteoblasts, maintain a constant bone mass throughout life. One of the mechanisms of non-traumatic ONFH is the increased bone resorption and/or decreased bone formation [2–4]. Osteoblasts are involved in bone matrix degradation and synthesis of collagen type I, a specific type of collagen that is synthesized by osteoblasts in bone tissue, and plays an important role in osteoblastic differentiation of BMSCs [5–8]. In addition, collagen type I is a major component of the bone extracellular matrix, and correlates with bone resorption [9, 10]. Thus, it has been suggested that collagen type I may be associated with an increased risk of ONFH.

The matrix metalloproteinase (MMP) family comprises 23 protein members, which have the ability to degrade virtually all structural components of the extracellular matrix. MMPs are thought to be the key regulators of tissue degradation and remodeling [11, 12]. Furthermore, MMPs are considered to play an important role in the regulation of osteogenesis and bone remodeling during embryogenesis, adolescence, and bone healing [13]. Matrix metalloproteinase-8 (also known as neutrophil collagenase and collagenase-2), encoded by *MMP8*, is produced predominantly in the bone marrow, and expressed in neutrophils and macrophages [14]. Up to now, two main roles of MMP-8 have been identified. First, MMP-8 can cleave the triple helix structure of native collagen, thus initiating the first steps of collagen degradation [15]. Second, since MMP-8 is highly expressed in neutrophils, increased expression and activity of MMP-8 can reflect neutrophils (associated with the initial inflammatory stages of wound repair) in the fluids of healing wounds, and can influence the wound healing [14, 16]. MMP-8 has the substrate specificity for interstitial collagen type I disruption [17]. In addition, increased expression of MMP-8 can promote osteoclast differentiation and activity [18]. Thus, we hypothesized that MMP-8 plays an important role in the pathogenesis of ONFH.

Recent studies have indicated that single nucleotide polymorphisms (SNPs) are associated with an increased risk of ONFH [19–22]. The polymorphisms of *MMP8* have been investigated in many diseases. In this case-control study, we genotyped five SNPs in *MMP8 (*rs3740938, rs2012390, rs1940475, rs11225394, and rs11225395) that have been associated with refractive error [23], sepsis [24], atherosclerosis [25], thoracic aortic dissection [26], bladder cancer [27], breast cancer [28], and steroid-induced ONFH [29]. We performed a comprehensive association analysis that indicates the association between these SNPs and ONFH risk in Chinese Han population.

## RESULTS

A total of 1092 participants (585 patients with ONFH and 507 healthy controls) were genotyped. The ONFH cases and healthy controls were matched in sex (P=0.293). Primers and PCR product sequences are shown in Table 1. Basic characteristics, including gender and age, are listed in Table 2. Table 3 summarizes the basic characteristics of SNPs in the study specimens. None of the tSNPs that were excluded deviated from Hardy–Weinberg equilibrium (HWE). The χ^2^ test was used to compare the differences in frequency distributions of alleles between cases and controls. One tSNP was associated with an increased risk of developing ONFH: rs11225394 in *MMP8* (OR=1.34; 95% CI, 1.003-1.786, P=0.047). The other SNPs did not correlate with the risk of ONFH.

**Table 1:** Primers Used for this Study

SNP_ID	1st-PCRP	2nd-PCRP	UEP_SEQ
rs3740938	ACGTTGGATGGTCAGTAAGAGGAATCAAAG	ACGTTGGATGTGACATTTGATGCTATCAC	GATGCTATCACCACACT
rs2012390	ACGTTGGATGACTGTTTCTAGGTCACACCC	ACGTTGGATGTCAGGGAGAGGAAGCAATTC	GAAGCAAATGTGAGGAAGAT
rs1940475	ACGTTGGATGTTTGGGTTGAATGTGACGGG	ACGTTGGATGTAAAACCACCACTGTCAGGC	CTCCACAGCGAGGCTTTT
rs11225394	ACGTTGGATGCAATCTCAAACTAATCACCC	ACGTTGGATGTTAGGAAATAGTGTGGGTTG	AGTGTGGGTTGTTTTCTCTT
rs11225395	ACGTTGGATGAGAGCTGCTGCTCCACTATG	ACGTTGGATGGTTTAGAGAGACTGAGCTGG	GCTGAGCTGGGAGCTACTATA

**Table 2:** Characteristics of cases and controls in this study

Various	casesn=585	Controlsn=507	P value
Sex			0.293^a^
male	472(80,7%)	396(78.1%)	
female	113(19.3%)	111(21.9%)	
Age, year (mean ± SD)	42.61±12.951	47.43±9.739	< 0.001 ^b^

*p* ≤ 0.05 indicates statistical significance

a Two-sided Chi-squared test

b Independent samples *t* test

**Table 3:** Allele frequencies in cases and controls and odds ratio estimates for ONFH

SNP	Gene	Locus	Alleles (A /B)	MAF		HWE *p*^a^ value	ORs	95%CI	*p*^b^ value
				Case	Control				
rs3740938	MMP8	11q22.2	A/G	0.243	0.235	0.621	1.04	0.856-1.270	0.680
rs2012390	MMP8	11q22.2	G/A	0.276	0.276	0.912	1.00	0.827-1.204	0.981
rs1940475	MMP8	11q22.2	T/C	0.387	0.369	0.775	1.08	0.909-1.286	0.378
rs11225394	MMP8	11q22.2	T/C	0.112	0.086	0.563	1.34	1.003-1.786	0.047*
rs11225395	MMP8	11q22.2	A/G	0.379	0.360	0.773	1.08	0.910-1.290	0.367

SNP single nucleotide polymorphism, HWE Hardy-Weinberg equilibrium, OR odds ratio, 95% CI 95% confidence interval, MAF minor allele frequency

* *p* ≤ 0.05 indicates statistical significance

a *p* was calculated by exact test

b *p* was calculated by Pearson Chi-squared test

Next, we assumed that the minor allele of each tSNP was a risk factor, and we assessed the association between these SNPs and ONFH risks using five genetic models (codominant, dominant, recessive, over-dominant, and log-additive) by unconditional logistic-regression analysis. Our analyses in Table 4 showed that the genotype “T/C” of rs11225394 in the *MMP8* gene was associated with an increased risk of ONFH in the dominant model before (OR =1.39, 95% CI, 1.02–1.89, P=0.036) and after (adjusted OR =1.44, 95% CI, 1.05–1.96, P=0.023) adjustment. Similarly, in the over-dominant model, rs11225394 also exhibited a significant association with the ONFH risk before (OR=1.39, 95% CI, 1.02–1.89, P=0.038) and after (OR =1.44, 95% CI, 1.05–1.98, P=0.022) adjustment. In addition, in the log-additive model, the T/C genotype of rs11225394 also conferred an increased risk before (OR =1.36, 95% CI, 1.01–1.84, P=0.039) and after (OR =1.40, 95% CI, 1.04–1.90, P=0.027) adjustment for age and sex.

**Table 4:** Genotypic model analysis of relationship between SNPs and ONFH risk

Model	Genotype	Group=control	Group=Hormone	Without Adjustment		With Adjustment		AIC	BIC
				OR (95% CI)	*p^a^*-value	OR (95% CI)	*p^a^*-value		
Codominant	C/C	405 (83.2%)	456 (78.1%)	1		1			
	T/C	80 (16.4%)	125 (21.4%)	**1.39 (1.02-1.89)**	0.11	**1.44 (1.05-1.98)**	0.073	1437.9	1462.8
	T/T	2 (0.4%)	3 (0.5%)	1.33 (0.22-8.01)		1.14 (0.18-7.08)			
Dominant	C/C	405 (83.2%)	456 (78.1%)	1	0.036	1	0.023	1435.9	1455.9
	T/C-T/T	82 (16.8%)	128 (21.9%)	**1.39 (1.02-1.89)**		**1.44 (1.05-1.96)**			
Recessive	C/C-T/C	485 (99.6%)	581 (99.5%)	1	0.8	1	0.95	1441.1	1461
	T/T	2 (0.4%)	3 (0.5%)	1.25 (0.21-7.52)		1.07 (0.17-6.61)			
Overdominant	C/C-T/T	407 (83.6%)	459 (78.6%)	1	0.038	1	0.022	1435.9	1455.8
	T/C	80 (16.4%)	125 (21.4%)	**1.39 (1.02-1.89)**		**1.44 (1.05-1.98)**			
Log-additive	---	---	---	**1.36 (1.01-1.84)**	0.039	**1.40 (1.04-1.90)**	0.027	1436.3	1456.2

* *p* ≤ 0.05 indicates statistical significance

*p* values were calculated by Wald test by unconditional logistic regression adjusted for age and gender

## DISCUSSION

In this study, five SNPs in the *MMP8* gene were examined in 1092 subjects to determine whether they were associated with the risk of ONFH in the Chinese Han population. The most valuable finding is that the rs11225394 polymorphism in *MMP8* showed a significant association with an increased risk of ONFH occurrence. We are the first to demonstrate an association between this locus and ONFH susceptibility.

Rs11225394 is located in the intron (boundary) region of the *MMP8* gene. Morgan *et al*. [30] has demonstrated that rs11225394 is associated with increased risk of ulcerative colitis. In addition, rs11225394 has been associated with the refractive error [31]. However, rs11225394 did not correlate with the risk of steroid-induced ONFH in the population of northern China and rs3740938, rs3740938, rs1940475, rs11225395 were associated with an increased risk of steroid-induced ONFH in the population of northern China [29]. This is inconsistent with our results, and this inconsistency may be explained by the genetic differences between steroid-induced ONFH and other forms of ONFH. These data illustrate the complexity of the mechanisms and multiple interacting networks involved in the SNPs, which may promote ONFH.

Like most MMPs, MMP-8 is secreted as an inactive pro-enzyme, which can be converted into an active protease [32]. The expression of MMP-8 is related to inflammatory cytokines, growth factors, and hormones [33]. In terms of orthopedic diseases and related disorders, MMP-8 is expressed in cells of the osteoblastic lineage, the mesenchymal condensation, periosteal cells, osteoblasts, osteocytes, muscle cells, and chondrocytes. The possible reason might be that MMP-8 may be involved in remodeling of collagenous ECMs during embryonic bone development [34]. In periodontitis, pro-inflammatory cytokines can stimulate gingival fibroblasts to secrete MMP-8, thereby enhancing ECM and basement membrane breakdown, which is one of the possible mechanisms causing periodontitis [18]. In osteosarcoma, *MMP8* is negatively regulated by microRNA-539 through a special binding site in the *MMP8* 3′-UTR, which may inhibit osteosarcoma cell proliferation and migration [35]. In carious dentin of primary teeth, a strong expression of MMP-8 was also observed in both active caries lesion and sealing infected caries dentin [36]. In arthritis, lack of MMP-8 is accompanied by exacerbated joint inflammation and bone erosion, indicating that MMP-8 might have a protective role in arthritis [37]. However, there is a limited evidence for a direct function of MMP-8 in orthopedic diseases. Currently, the relationship between rs11225394 polymorphism and *MMP8* gene expression/function in ONFH patients is not clear. Further studies are required to characterize the function of *MMP8* and elucidate the mechanisms underlying the association between *MMP8* and ONFH susceptibility.

Our study provides the first evidence of the association between rs11225394 in *MMP8* and the risk of ONFH. Although this study had a sufficient statistical power, there were some intrinsic limitations. First, the participants’ ethnicity was limited to the Han Chinese population. Thus, further analysis is needed to determine whether current conclusions are applicable also to other ethnicities. Second, the participant cases were enrolled in the same hospital, so selection bias cannot be excluded and the subjects might not be representative of the general population. However, this bias was not meaningful because the samples did not differ in geographical distributions or genotype frequencies. Finally, even though we discovered the association between rs11225394 and ONFH susceptibility, we did not elucidate causal mechanisms.

Together, our results provide the first evidence regarding the relationship between *MMP8* and the risk of ONFH. We believe that our results will encourage further studies to characterize the function of *MMP8* and elucidate the underlying mechanisms of *MMP8* polymorphisms conferring susceptibility to ONFH.

## MATERIALS AND METHODS

### Study participants and data collection

In this case-control study, we recruited participants among Chinese population including patients with confirmed ONFH and with the mean age of 42.61±12.951, as well as healthy controls with the mean age of 47.43±9.739. These cases were recruited from the Zhengzhou Traditional Chinese Medicine (TCM) Traumatology Hospital between January 2013 and May 2015. Control subjects were genetically unrelated Chinese residents who were enrolled from the Zhengzhou Medical Center in Henan Province. All participants were Han Chinese. A standard epidemiologic questionnaire was used to collect personal data. Informed consent was obtained from all participants of the study. The protocols for were approved by the Zhengzhou TCM Traumatology Hospital Human Research Committee for Approval of Research Involving Human Subjects.

### Inclusion and exclusion criteria

The diagnosis of ONFH was defined according to the following criteria: (1) clinical ONFH manifestations, such as pain and activity limitation of hip, sick side lower limb muscle atrophy. (2) MRI and plain radiography changes can yield the most accurate diagnosis like high density shadows of femoral head, hip joint narrowness and bumpiness, or joint surface rupture. (3) All patients had a physical examination. Those who met the criteria for ONFH were selected. Patients who were diagnosed by MRI without abnormalities on plain radiography were also selected for this study. Patients with an explicit history of direct trauma or with possible combined causes were excluded. Those who had a chronic metabolic disorder of heart, kidney, or liver were also excluded.

All control subjects were healthy. Selection criteria were the following: (1) No hip pain; (2) Anteroposterior and frog-leg lateral pelvic radiographs did not show any lesions; (3) Subjects with a long term history of alcohol and steroid use were excluded; and (4) All individuals related to the enrolled patients were excluded from the control group.

### Selection of single-nucleotide polymorphisms and genotyping methods

All five primers from one gene were designed to amplify fragments of rs3740938, rs3740938, rs1940475, rs11225394, and rs11225395. In our ongoing case-control study, 5 ml of peripheral blood were drawn into coded sodium citrate-coated tubes from each participant and stored at –80°C after centrifugation. Extraction of DNA from whole blood samples was performed using the GoldMag-Mini Whole Blood Genomic DNA Purification Kit (GoldMag Co. Ltd. Xi’an City, China) and the DNA concentration was measured by NanoDrop 2000 spectrophotometer. The Multiplexed SNP MassEXTENDED assay was designed by Sequenom MassARRAY Assay Design 3.0 Software. Genotyping was performed using the Sequenom MassARRAY RS1000 system according to the manufacture's protocol. Data management and analysis were performed using Sequenom Typer 4.0 Software.

### Statistical analysis

Data were analyzed by the SPSS version 18.0 statistical software (SPSS, Chicago, IL) and Microsoft Excel. We performed the 2-side chi-square tests to estimate the genotype frequencies of case and control individuals; a P-value of less than 0.05 was considered statistically significant. A Fisher's exact test was used to assess the departure of each SNP frequency from Hardy–Weinberg equilibrium (HWE) in the control subjects. ORs and 95%CIs were determined using unconditional logistic regression analysis with adjustments for age and sex. The relation between the *MMP8* gene and the risk of ONFH was tested in dominant and recessive models and also codominant, over-dominant and log-additive effects. We used Akaike's Information Criterion and Bayesian Information Criterion to estimate the best-fit model for each SNP.
